# Species composition, community and population dynamics of two gallery forests from the Brazilian Cerrado domain

**DOI:** 10.3897/BDJ.4.e8503

**Published:** 2016-07-18

**Authors:** Markus Gastauer, Roosevelt P Almado, Angela S Miazaki, Écio S Diniz, Luis C B Moreira, João A.A. Meira-Neto

**Affiliations:** ‡ Laboratory of Plant Ecology and Evolution, Department of Plant Biology, Federal University of Viçosa, Viçosa, Brazil; § ArcelorMittal Bioflorestas, Belo Horizonte, Brazil; | State University of Minas Gerais, Campus Frutal, Frutal, Brazil

**Keywords:** Gallery Forest, Brazilian Cerrado, savanna vegetation, species richness, tropical forests, forest inventory

## Abstract

**Background:**

To understand the impacts of global changes on future community compositions, knowledge of community dynamics is of crucial importance. To improve our knowledge of community composition, biomass stock and maintenance of gallery forests in the Brazilian Cerrado, we provide two datasets from the 0.5 ha Corrego Fazendinha Gallery Forest Dynamics Plot and the Corrego Fundo Gallery Forest Dynamics Plot situated in the Bom Despacho region, Minas Gerais, Southeastern Brazil.

**New information:**

We report diameter at breast height, basal area and height measurements of 3417 trees and treelets identified during three censuses in both areas.

## Introduction

Although the Brazilian Cerrado is a hotspot of biodiversity (Mendonça et al. 2008, Myers et al. 2000) and holds carbon stocks of nearly 300 Mg per hectare (Batlle-Bayer et al. 2010, Paiva et al. 2011), its species richness, diversity and biomass are still threatened by habitat loss, fragmentation, biological invasion and climate change (e.g., Lapola et al. 2013, Jantz et al. 2015, Rossi et al. 2014). Within the Cerrado domain, gallery forests accompany the borders of rivers, creeks and streams, forming important corridors for wildlife among patches of remaining vegetation (Silveira et al. 2014) that also protect aquatic ecosystems from substrate input, reducing water temperatures and erosion of river banks (Monteiro et al. 2016, Londe and Silva 2014). Furthermore, gallery forests have the highest above ground biomass per hectare in the Cerrado domain (Moreira-Burger and Delitti 1999). Worldwide, these forests are threatened by human activities, including domestic livestock, which prevent tree seedling establishment, and the construction of dams and weirs, which cause flooding or interference with natural stream flow (FAO 2010). Long-term monitoring studies, so-called community dynamics, are necessary to outline and understand the impacts of these disturbances on vegetation communities and on carbon stocks (Couvet et al. 2011, Fidelis et al. 2012, Pocock et al. 2015).

Therefore, the aim of this data paper is to make available data from forest dynamics from two gallery forest dynamics plots from the Bom Despacho region, Minas Gerais, Southeastern Brazil, to increase knowledge about community composition, biomass stock and maintenance of such forests in the Brazilian Cerrado.

## Project description

### Title

Population and community dynamics of two gallery forests from the Bom Despacho Region, Minas Gerais, Brazil

### Study area description

The study was carried out in the counties of Quartel Geral and Dores do Indaía, Bom Despacho region, Minas Gerais, Brazil. Cattle pasture, corn and eucalyptus plantations characterize the land-use of both counties. According to the Köppen system, the climate is humid subtropical (Cwa, Peel et al. 2007), with warm and moist conditions in the summer months, dry winters and an annual precipitation of approximately 1,170 mm. The predominant soils are deeply weathered latosols. According to Veloso et al. (1991), the vegetation is characterized as savanna vegetation.

**Figure 1.**
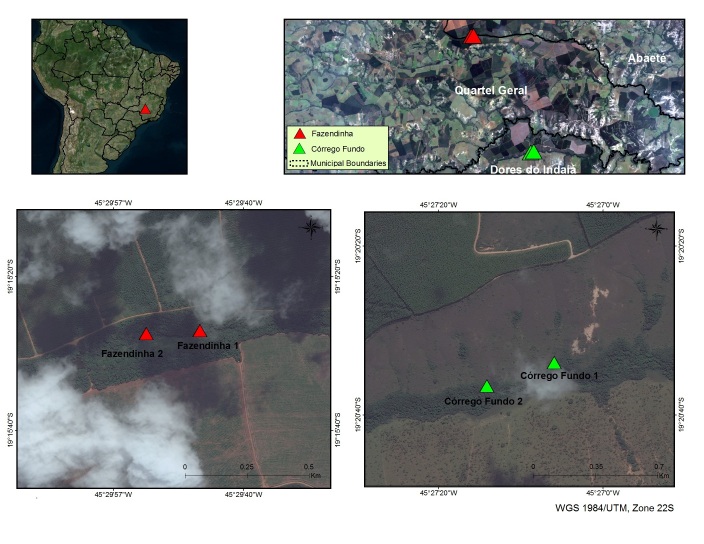
Localization of study sites

Within the municipality, two study sites were selected within properties owned by the ArcelorMittal Bioflorestas company. The Corrego Fazendinho Gallery Forest, situated 5.5 km west of Quartel Geral center, covers approximately 50 ha on both sides of the upper 5 km of Fazendinho Creek (Fig. 1). It is surrounded on all sides by eucalypt plantations from ArcelorMittal Bioflorestas.

The second study site, the Corrego Fundo Gallery Forest, is situated approximately 10 km southeast of the Corrego Fazendinho Gallery Forest (Fig. 1). It is a forest remnant that flanks the complete upper Fundo Creek. The mean width of the gallery forest is approximately 80 m. On its northern side, the forest adjoins native Cerrado vegetation belonging to the ArcelorMittal Bioflorestas legal reserve, while cattle pastures are found beyond its southern limit.

### Funding

JAAMN received a CNPq productivity fellowship. ArcelorMittal Bioflorestas, FAPEMIG and CNPq financed this study.

## Sampling methods

### Sampling description

Within each of the gallery forests, two plots of 50 x 50 m were delimitated and divided into 25 subplots of 10 × 10 m (24 plots in the second plot from the Corrego Fundo Gallery Forest). All plots are situated at the northern part of the gallery forests (see Fig. 1).

Within these plots, three censuses in four-year intervals of all trees with a diameter at breast height (dbh) greater than 3.2 cm were carried out (Table 1). Trees fulfilling the inclusion criterion were tagged and identified. Tree diameter (dbh) was measured and basal area was calculated; for multiple stem individuals, we calculated basal area at breast height for all shoots, summed these, and calculated from that the pooled dbh.

Specimens not recognized during fieldwork were collected, deposited in the Herbarium of the Federal University of Viçosa (VIC) and identified with the help of material from the VIC or by consultation of specialists and literature sources (Lorenzi 1992). Species names were verified using the Taxonomic Name Resolution Service (TNRS) proposed by Boyle et al. (2013); species classification follows the Angiosperm Phylogeny Group III guidelines (APG III 2009).

Diversity indices as well as Jaccard similarity between study sites were computed using EstimateS (Colwell and Coddington 1994). Mortality and recruitment rates, as well as gains and losses of the basal area, were calculated according to Sheil (1995).

## Geographic coverage

### Description

This study was carried out in the Counties Quartel Geral and Dores do Indaía, Bom Despacho region, Minas Gerais, Brazil (Fig. 1).

### Coordinates

-19.343498 and -19.2575 Latitude; -45.49603 and -45.451609 Longitude.

## Taxonomic coverage

### Description

Altogether, 3413 trees and treelets belonging to 158 species, 96 genera and 41 families were detected in both study sites during all censuses. Thirty species were identified to genus level only, two to family level, and three species remain unidentified.

With a total of 1862 trees and treelets from 114 species (70 genera, 35 families) from three censuses, species richness and stem density in the Corrego Fazendinha Gallery Forest was higher than in the Corrego Fundo Gallery Forest (1551 trees and treelets, 89 species, 67 genera, 35 families, Table 1). Forty-five species occur in both study sites, yielding a Jaccard similarity between the study sites of 0.28. More than 75% of species and around 80% of basal area belongs to common species.

Due to higher species richness, diversity is also higher in the Corrego Fazendinha Gallery Forest than in the Corrego Fundo Gallery Forest. While the basal area increased from the first to the third census in the Corrego Fazendinha Gallery Forest, it declined in the Corrego Fundo Gallery Forest (Tables 1, 2).

With regards to basal area and abundance, Vochysiaceae, Fabaceae, Myrtaceae, Lauraceae and Anacaridaceae are among the five dominant families in the Corrego Fazendinha and Corrego Fundo gallery forests (Tables 3, 4). Furthermore, Fabaceae, Myrtaceae and Anacardiaceae are the most species-rich families in both study sites. *Callisthene*, *Myrcia* and *Copaifera* had the highest basal area among genera, while *Callisthene*, *Siparuna* and *Myrcia* showed the highest abundance. Finally, *Myrcia*, *Machaerium* and *Aspidosperma* are the most species-rich genera in both study sites (Tables 5, 6). *Callisthene major* and *Copaifera langsdorffii* have the highest basal area in both study sites, followed by *Piptadenia gonoacantha*, *Siparuna guinanesis* and *Myrcia tomentosa* in the Corrego Fazendinha Gallery Forest and *Terminalia glabrescens*, *Tapirira guianensis* and *Pera glabrata* in the Corrego Fundo Gallery Forest. The most abundant species in both study sites are *C. major* and *S. guianensis*, followed by *Campomanesia xanthocarpa*, *Myrcia tomentosa* and *Dalbergia brasiliensis* in the Corrego Fazendinha Gallery Forest and *Licania kunthiana*, *Myrcia rostrata* and *Alibertia edulis* in the Corrego Fundo Gallery Forest (Tables 7, 8).

The recruitment rate in the Corrego Fazendinha Gallery Forest exceeded the mortality rate during 2007 and 2011; but mortality was higher than recruitment in the period from 2011 to 2015 (Table 9). In the Corrego Fundo Gallery Forest, mortality exceeded recruitment during both observed periods. Further, gains of basal area were higher than losses in the Corrego Fazendinha Gallery Forest, indicating an increase in carbon stock, while losses in the Corrego Fundo Gallery Forest outpaced its gains (Table 9).

Although both gallery forests are situated in the same region, they show low similarity between them, indicating high beta-diversity of this ecosystem, which might be due to high environmental heterogeneity (Chave 2009), different disturbance regimes (Connell 1978, Mendonça Machado and de Oliveira-Filho 2010), differences in successional stages (Magurran 2011) or neutral factors such as ecological drift and stochasticity (Hubbell 2001). Large numbers of individuals and basal area belonging to common species indicates that the high beta-diversity is due to large number of species represented by few individuals only, which is typical for tropical forests (Condit 2000). High beta-diversity increases the importance for the protection of biotic ressources and highlights the demand for further research to understand underlying determinants.

Although study sites were sampled three times during similar periods, forest dynamics show large differences between study sites. Mortality and recruitment rates between 1.5 and 3 % are within the expectations for undisturbed alluvial or gallery forests (Higuchi et al. 2008, Fontes and Teles Walter 2011). Causes for the elevated mortality rate during the second observation period in Corrego Fazendinha Gallery Forest remain unknown, as external disturbances were not registered during the field campaigns, but may be related to extreme water deficits between 2012 and 2015 in Brazil (Getirana 2016). These findings indicate the importance to give once continuity and to amplify these kind of studies, to come to a better understanding of the drivers of forest dynamics that influence the maintanence of biodiversity as well as that ecosystem services such as carbon sequestration in biomass.

### Taxa included

**Table:** 

Rank	Scientific Name	
species	Acrocomia aculeata	
subspecies	Alchornea glandulosa subsp. iricurana	
species	Alibertia edulis	
species	Andira fraxinifolia	
species	Apeiba tibourbou	
species	Apuleia leiocarpa	
species	Ardisia glauciflora	
species	Aspidosperma cylindrocarpon	
species	Aspidosperma darienense	
species	Aspidosperma olivaceum	
genus	Aspidosperma sp1	
genus	Aspidosperma sp2	
species	Aspidosperma subincanum	
species	Astronium fraxinifolium	
genus	Astronium sp.	
species	Aureliana velutina	
species	Banisteriopsis anisandra	
species	Bowdichia virgilioides	
species	Brosimum gaudichaudii	
species	Byrsonima sericea	
species	Callisthene major	
species	Calophyllum brasiliense	
genus	Calyptranthes sp.	
genus	Campomanesia sp.	
species	Campomanesia velutina	
species	Campomanesia xanthocarpa	
species	Casearia gossypiosperma	
genus	Casearia sp.	
species	Casearia sylvestris	
species	Cassia ferruginea	
genus	Cassia sp.	
species	Cecropia hololeuca	
species	Cecropia pachystachya	
species	Cedrela fissilis	
species	Copaifera langsdorffii	
species	Cupania vernalis	
species	Dalbergia brasiliensis	
species	Dalbergia frutescens	
species	Dendropanax cuneatus	
genus	Dendropanax sp.	
genus	Dialium sp.	
species	Dilodendron bipinnatum	
species	Diospyros brasiliensis	
species	Diospyros hispida	
genus	Diospyros sp.	
species	Duguetia lanceolata	
species	Endlicheria paniculata	
species	Eriotheca candolleana	
species	Erythroxylum citrifolium	
species	Erythroxylum daphnites	
species	Erythroxylum pelleterianum	
species	Eugenia dodonaeifolia	
species	Eugenia dysenterica	
species	Eugenia florida	
genus	Eugenia sp.	
species	Guapira opposita	
genus	Guarea sp.	
species	Guatteria sellowiana	
species	Guazuma ulmifolia	
species	Guettarda viburnoides	
species	Handroanthus ochraceus	
species	Heteropterys byrsonimifolia	
species	Hirtella hebeclada	
species	Ilex cerasifolia	
genus	Ilex sp.	
species	Ixora gardneriana	
species	Lacistema hasslerianum	
genus	Lacistema sp.	
species	Licania kunthiana	
genus	Licania sp.	
species	Lithraea molleoides	
species	Luehea grandiflora	
species	Machaerium brasiliense	
species	Machaerium isadelphum	
species	Machaerium nyctitans	
species	Machaerium opacum	
genus	Machaerium sp.	
species	Machaerium villosum	
species	Maclura tinctoria	
genus	Marlieria sp.	
species	Matayba floribunda	
species	Matayba guianensis	
genus	Maytenus sp.	
species	Micropholis gardneriana	
species	Myracrodruon urundeuva	
species	Myrcia guianensis	
species	Myrcia lingua	
species	Myrcia multiflora	
species	Myrcia rostrata	
species	Myrcia rufipes	
genus	Myrcia sp.	
genus	Myrcia sp1	
genus	Myrcia sp2	
genus	Myrcia sp3	
species	Myrcia splendens	
species	Myrcia tomentosa	
species	Myrsine coriacea	
species	Myrsine umbellata	
family	Myrtaceae sp.	
species	Nectandra oppositifolia	
species	Ocotea corymbosa	
species	Ouratea castaneifolia	
genus	Ouratea sp.	
species	Peltophorum dubium	
species	Pera glabrata	
species	Picramnia parvifolia	
species	Piper arboreum	
species	Piptadenia gonoacantha	
species	Plathymenia reticulata	
species	Platypodium elegans	
species	Pouteria glomerata	
species	Protium heptaphyllum	
species	Pseudobombax tomentosum	
species	Psidium guajava	
species	Psidium rufum	
genus	Psidium sp.	
genus	Pterogyne sp.	
species	Qualea grandiflora	
species	Qualea multiflora	
subspecies	Qualea multiflora subsp. pubescens	
genus	Qualea sp.	
species	Randia armata	
species	Rollinia laurifolia	
genus	Rollinia sp.	
family	Rubiaceae sp.	
species	Rudgea viburnoides	
species	Salacia elliptica	
species	Schefflera morototoni	
species	Sclerolobium paniculatum	
species	Senna macranthera	
genus	Senna sp1	
genus	Senna sp2	
species	Simarouba amara	
species	Siparuna guianensis	
species	Sterculia striata	
species	Swartzia myrtifolia	
genus	Swartzia sp.	
species	Tabebuia impetiginosa	
species	Tabebuia serratifolia	
species	Tapirira guianensis	
species	Tapirira obtusa	
species	Terminalia argentea	
species	Terminalia glabrescens	
species	Trichilia pallida	
genus	Trichilia sp.	
genus	Vernonia sp.	
species	Virola sebifera	
species	Vitex polygama	
species	Vitex sellowiana	
species	Xylopia aromatica	
species	Xylopia sericea	
species	Xylosma prockia	
species	Zanthoxylum rhoifolium	
species	Zanthoxylum riedelianum	

## Usage rights

### Use license

Creative Commons Public Domain Waiver (CC-Zero)

### IP rights notes

This dataset can be freely used, provided this Data Paper is cited.

## Data resources

### Data package title

Species composition, community and population dynamics of two gallery forests from the Brazilian Cerrado domain

### Resource link

http://187.32.44.123/ipt/resource.do?r=fazendinha

### Alternative identifiers

http://www.gbif.org/publisher/9e1ad169-1f58-48fb-ad7a-3b2b4544d875

### Number of data sets

2

### Data set 1.

#### Data set name

Community Dynamics of Corrego Fazendinha Gallery Forest

#### Data format

Darwin Core Archive DwC-A

#### Number of columns

1

#### Download URL

http://187.32.44.123/ipt/resource.do?r=fazendinha, http://www.gbif.org/dataset/5ddd59c2-c291-4a74-8a25-933bf873d4a4

#### Description

Occurrences, basal area and height of 1862 trees and treelets identified during three census distributed within all 50 subplots from the 0.5 ha Corrego Fazendinha Gallery Forest Dynamics Plot, Quartel Geral, Minas Gerais, Brazil. Dataset (Suppl. material 1) consists of occurrence.txt (DwC-Attributes id, modified, language, rights, rightsHolder, bibliographicCitation, references, datasetName, basisOfRecord, occurrenceID, occurrenceRemarks, eventDate, decimalLatitude, decimalLongitude, acceptedNameUsageID, parentNameUsageID, nameAccordingToID, scientificName, acceptedNameUsage, parentNameUsage, nameAccordingTo, higherClassification, kingdom, class, order, family, genus, subgenus, specificEpithet, infraSpecificEpithet, taxonRank, scientificNameAuthorship, nomenclaturalCode, taxonomicStatus), meta.xml, measurementOrFact.txt (continaining the DwC-Attributes id, measurementType, measurementUnit, measurementDeterminedDate, measurementMethod, measurementValue, measurementRemarks, locationID), eml.xml, ressourcerelationship.txt (containing the DwC-Attributes id, locationID, resourceRelationshipIDresourceID, relatedResourceID, Role). Please see http://rs.tdwg.org/dwc/ for details.

**Data set 1.** 

Column label	Column description
id	Occurrence identifier

### Data set 2.

#### Data set name

Community Dynamics of Corrego Fundo Gallery Forest

#### Data format

Darwin Core Archive DwC-A

#### Number of columns

1

#### Download URL

http://www.gbif.org/dataset/a68403f2-b43f-4747-bd54-1e3eeb03dd46, http://187.32.44.123/ipt/resource.do?r=fundo

#### Description

Occurrences, basal area and height of 1551 trees and treelets identified during three census distributed within all 49 subplots from the 0.49 ha Corrego Fazendinha Gallery Forest Dynamics Plot, Quartel Geral, Minas Gerais, Brazil. Dataset (Suppl. material 2) consists of the occurrence.txt (DwC-Attributes id, modified, language, rights, rightsHolder, bibliographicCitation, references, datasetName, basisOfRecord, occurrenceID, occurrenceRemarks, eventDate, decimalLatitude, decimalLongitude, acceptedNameUsageID, parentNameUsageID, nameAccordingToID, scientificName, acceptedNameUsage, parentNameUsage, nameAccordingTo, higherClassification, kingdom, class, order, family, genus, subgenus, specificEpithet, infraSpecificEpithet, taxonRank, scientificNameAuthorship, nomenclaturalCode, taxonomicStatus), meta.xml, measurementOrFact.txt (continaining the DwC-Attributes id, measurementType, measurementUnit, measurementDeterminedDate, measurementMethod, measurementValue, measurementRemarks, locationID), eml.xml, ressourcerelationship.txt (containing the DwC-Attributes id, locationID, resourceRelationshipIDresourceID, relatedResourceID, Role). Please see http://rs.tdwg.org/dwc/ for details.​

**Data set 2.** 

Column label	Column description
id	Occurrence identifier

## Supplementary Material

Supplementary material 1 Community Dynamics of Corrego Fazendinha Gallery Forest Data type: Darwin Core Archive
File: oo_81543.zip Markus Gastauer, Roosevelt de Paula Almado, Angela S. Miazaki, Écio D. Souza, Luiz C.B. Moreira & João A. A. Meira-Neto

Supplementary material 2 Community Dynamics of Corrego Fundo Gallery Forest Data type: Darwin Core Archive
File: oo_81544.zip Markus Gastauer, Roosevelt de Paula Almado, Angela S. Miazaki, Écio D. Souza, Luiz C.B. Moreira & João A. A. Meira-Neto

## Figures and Tables

**Table 1.** Gallery forest plots census histories. BA is basal area.

**Census**	**Dates**	**BA [m^2^**]	**Number of trees**	**Number of species**	**BA ≥ 10 cm [m^2^**]	**Number of trees ≥ 10 cm**	**Number of species (≥ 10 cm dbh)**
Corrego Fazendinha Gallery Forest							
First	June 2007	12.61	1597	110	9.35	405	67
Second	August 2011	14.06	1711	113	10.70	428	69
Third	August 2015	14.85	1478	101	11.93	434	66
Corrego Fundo Gallery Forest							
First	July 2006	13.02	1427	85	10.17	318	48
Second	August 2010	11.98	1374	90	9.16	317	48
Third	August 2014	11.34	1268	88	8.58	288	51

**Table 2.** Gallery forest plots diversity and species richness summary Tally (third censuses). N is number of individual trees, S is number of species, G is number of genera, F is number of families, H‘ is Shannon-Wiener diversity index using log_10_, and α is Fisher´s α. Basal area (BA) includes all multiple stems for each individual.

**Size Class [cm dbh**]	**BA [m^2^**]	**N**	**S**	**G**	**F**	**H**‘	**α ±DP**
Corrego Fazendinha Gallery Forest							
≥ 3.2	14.85	1478	101	64	31	3.41	24.54 ±1.29
≥ 10	11.93	434	66	48	26	3.12	21.66 ±1.75
≥ 30	2.86	26	9	9	7	1.83	4.87 ±1.52
Corrego Fundo Gallery Forest							
≥ 3.2	11.34	1268	88	64	35	3.43	21.49 ±1.21
≥ 10	8.61	292	51	40	23	3.02	17.87 ±1.72
≥ 30	3.46	25	5	5	5	1.10	1.87 ±0.61

**Table 3.** Corrego Fazendinha Gallery Forest rankings by family according to basal area (BA, including all multiple stems for each individual), number of individuals (N) and number of species (S), data from the third census.

**Rank**	**Family**	**BA**	% **BA**	% **N**	**Family**	**N**	% **N**	**Family**	**S**
1	Vochysiaceae	5.21	35.08	22.73	Vochysiaceae	336	22.73	Fabaceae	21
2	Fabaceae	3.08	20.76	13.53	Siparunaceae	274	18.54	Myrtaceae	16
3	Myrtaceae	1.59	10.74	16.24	Myrtaceae	240	16.24	Anacardiaceae	6
4	Lauraceae	0.91	6.12	4.80	Fabaceae	200	13.53	Rubiaceae	6
5	Anacardiaceae	0.79	5.31	4.06	Lauraceae	71	4.80	Annonaceae	5
6	Siparunaceae	0.65	4.40	18.54	Anacardiaceae	60	4.06	Vochysiaceae	5
7	Meliaceae	0.46	3.12	0.61	Aquifoliaceae	48	3.25	Apocynaceae	4
8	Annonaceae	0.42	2.83	2.77	Annonaceae	41	2.77	Araliaceae	3
9	Aquifoliaceae	0.21	1.44	3.25	Myristicaceae	36	2.44	Meliaceae	3
10	Salicaceae	0.21	1.43	1.15	Araliaceae	27	1.83	Aquifoliaceae	2
11	Combretaceae	0.17	1.13	0.41	Lacistemataceae	19	1.29	Chrysobalanaceae	2
12	Araliaceae	0.14	0.96	1.83	Rubiaceae	18	1.22	Combretaceae	2
13	Myristicaceae	0.12	0.80	2.44	Salicaceae	17	1.15	Erythroxylaceae	2
14	Arecaceae	0.09	0.63	0.14	Chrysobalanaceae	10	0.68	Euphorbiaceae	2
15	Chrysobalanaceae	0.09	0.60	0.68	Meliaceae	9	0.61	Lacistemataceae	2
16	Asteraceae	0.08	0.55	0.41	Malvaceae	7	0.47	Lauraceae	2
17	Picramniaceae	0.08	0.55	0.14	Apocynaceae	6	0.41	Malvaceae	2
18	Lamiaceae	0.08	0.53	0.20	Asteraceae	6	0.41	Not identified	2
19	Euphorbiaceae	0.08	0.51	0.34	Bignoniaceae	6	0.41	Arecaceae	1
20	Bignoniaceae	0.07	0.47	0.41	Combretaceae	6	0.41	Asteraceae	1
21	Malvaceae	0.07	0.45	0.47	Piperaceae	6	0.41	Bignoniaceae	1
22	Rubiaceae	0.06	0.40	1.22	Burseraceae	5	0.34	Burseraceae	1
23	Sapindaceae	0.04	0.24	0.34	Erythroxylaceae	5	0.34	Ebenaceae	1
24	Apocynaceae	0.04	0.24	0.41	Euphorbiaceae	5	0.34	Lamiaceae	1
25	Lacistemataceae	0.02	0.16	1.29	Sapindaceae	5	0.34	Malpighiaceae	1
26	Burseraceae	0.02	0.13	0.34	Not identified	4	0.27	Myristicaceae	1
27	Not identified	0.02	0.11	0.27	Lamiaceae	3	0.20	Ochnaceae	1
28	Erythroxylaceae	0.01	0.09	0.34	Arecaceae	2	0.14	Picramniaceae	1
29	Ebenaceae	0.01	0.06	0.07	Malpighiaceae	2	0.14	Piperaceae	1
30	Piperaceae	0.01	0.05	0.41	Picramniaceae	2	0.14	Salicaceae	1
31	Ochnaceae	0.01	0.05	0.07	Ebenaceae	1	0.07	Sapindaceae	1
32	Malpighiaceae	0.01	0.04	0.14	Ochnaceae	1	0.07	Siparunaceae	1

**Table 4.** Corrego Fundo Gallery Forest rankings by family according to basal area (BA, including all multiple stems for each individual), number of individuals (N) and number of species (S), data from the third census.

**Rank**	**Family**	**BA**	%**BA**	%**N**	**Family**	**N**	%**N**	**Family**	**S**
1	Vochysiaceae	3.78	33.30	2.63	Vochysiaceae	240	18.93	Myrtaceae	14
2	Fabaceae	1.53	13.49	1.06	Myrtaceae	148	11.67	Fabaceae	12
3	Combretaceae	0.77	6.82	0.54	Siparunaceae	132	10.41	Malvaceae	6
4	Anacardiaceae	0.76	6.67	0.53	Fabaceae	88	6.94	Rubiaceae	6
5	Myrtaceae	0.75	6.57	0.52	Chrysobalanaceae	84	6.62	Anacardiaceae	4
6	Malvaceae	0.48	4.26	0.34	Rubiaceae	69	5.44	Apocynaceae	4
7	Euphorbiaceae	0.47	4.12	0.32	Anacardiaceae	63	4.97	Sapindaceae	4
8	Sapindaceae	0.35	3.12	0.25	Lacistemataceae	49	3.86	Chrysobalanaceae	3
9	Burseraceae	0.32	2.85	0.22	Sapindaceae	49	3.86	Salicaceae	3
10	Siparunaceae	0.31	2.72	0.21	Burseraceae	39	3.08	Annonaceae	2
11	Apocynaceae	0.27	2.39	0.19	Combretaceae	34	2.68	Araliaceae	2
12	Chrysobalanaceae	0.27	2.39	0.19	Euphorbiaceae	33	2.60	Bignoniaceae	2
13	Annonaceae	0.26	2.32	0.18	Myristicaceae	30	2.37	Lauraceae	2
14	Rubiaceae	0.24	2.15	0.17	Apocynaceae	26	2.05	Primulaceae	2
15	Myristicaceae	0.13	1.18	0.09	Ebenaceae	25	1.97	Sapotaceae	2
16	Ebenaceae	0.12	1.02	0.08	Malvaceae	25	1.97	Aquifoliaceae	1
17	Bignoniaceae	0.10	0.92	0.07	Annonaceae	24	1.89	Burseraceae	1
18	Araliaceae	0.09	0.77	0.06	Araliaceae	19	1.50	Calophyllaceae	1
19	Lacistemataceae	0.07	0.61	0.05	Bignoniaceae	17	1.34	Celastraceae	1
20	Lamiaceae	0.05	0.43	0.03	Sapotaceae	12	0.95	Combretaceae	1
21	Sapotaceae	0.05	0.42	0.03	Aquifoliaceae	8	0.63	Ebenaceae	1
22	Salicaceae	0.03	0.26	0.02	Lamiaceae	8	0.63	Erythroxylaceae	1
23	Solanaceae	0.03	0.24	0.02	Salicaceae	8	0.63	Euphorbiaceae	1
24	Ochnaceae	0.02	0.22	0.02	Ochnaceae	7	0.55	Lacistemataceae	1
25	Lauraceae	0.01	0.13	0.01	Primulaceae	6	0.47	Lamiaceae	1
26	Aquifoliaceae	0.01	0.13	0.01	Lauraceae	5	0.39	Malpighiaceae	1
27	Primulaceae	0.01	0.11	0.01	Celastraceae	4	0.32	Meliaceae	1
28	Nyctaginaceae	0.01	0.10	0.01	Nyctaginaceae	4	0.32	Myristicaceae	1
29	Urticaceae	0.01	0.09	0.01	Solanaceae	4	0.32	Nyctaginaceae	1
30	Celastraceae	0.01	0.08	0.01	Calophyllaceae	2	0.16	Ochnaceae	1
31	Malpighiaceae	0.00	0.04	0.00	Malpighiaceae	2	0.16	Simaroubaceae	1
32	Erythroxylaceae	0.00	0.03	0.00	Erythroxylaceae	1	0.08	Siparunaceae	1
33	Calophyllaceae	0.00	0.02	0.00	Meliaceae	1	0.08	Solanaceae	1
34	Meliaceae	0.00	0.02	0.00	Simaroubaceae	1	0.08	Urticaceae	1
35	Simaroubaceae	0.00	0.01	0.00	Urticaceae	1	0.08	Vochysiaceae	1

**Table 5.** Corrego Fazendinha Gallery Forest ranking by genus according to basal area (BA, including all multiple stems for each individual), number of individuals (N) and number of species (S), data from the third census.

**Rank**	**Genus**	**BA**	% **BA**	%**N**	**Genus**	**N**	%**N**	**Genus**	**S**
1	*Callisthene* (Vochysiaceae)	5.066	34.12	21.11	*Callisthene* (Vochysiaceae)	312	21.11	*Myrcia* (Myrtaceae)	7
2	*Myrcia* (Myrtaceae)	1.003	6.75	7.24	*Siparuna* (Siparunaceae)	274	18.54	*Machaerium* (Fabaceae)	5
3	*Copaifera* (Fabaceae)	0.955	6.43	2.98	*Myrcia* (Myrtaceae)	107	7.24	*Aspidosperma* (Apocynaceae)	4
4	*Piptadenia* (Fabaceae)	0.686	4.62	1.08	*Campomanesia* (Myrtaceae)	96	6.50	*Pterogyne* (Fabaceae)	4
5	*Siparuna* (Siparunaceae)	0.654	4.40	18.54	*Dalbergia* (Fabaceae)	49	3.32	*Xylopia* (Annonaceae)	4
6	*Ocotea* (Lauraceae)	0.518	3.49	1.76	*Ilex* (Aquifoliaceae)	48	3.25	*Eugenia* (Myrtaceae)	3
7	*Machaerium* (Fabaceae)	0.462	3.11	1.96	*Nectandra* (Lauraceae)	45	3.04	*Astronium* (Anacardiaceae)	2
8	*Cedrela* (Meliaceae)	0.449	3.03	0.41	*Copaifera* (Fabaceae)	44	2.98	*Campomanesia* (Myrtaceae)	2
9	*Campomanesia* (Myrtaceae)	0.443	2.98	6.50	*Virola* (Myristicaceae)	36	2.44	*Cassia* (Fabaceae)	2
10	*Nectandra* (Lauraceae)	0.391	2.64	3.04	*Xylopia* (Annonaceae)	35	2.37	*Dendropanax* (Araliaceae)	2
11	*Xylopia* (Annonaceae)	0.355	2.39	2.37	*Swartzia* (Fabaceae)	32	2.17	*Erythroxylum* (Erythroxylaceae)	2
12	*Lithraea* (Anacardiaceae)	0.334	2.25	1.01	*Machaerium* (Fabaceae)	29	1.96	*Ilex* (Aquifoliaceae)	2
13	*Dalbergia* (Fabaceae)	0.273	1.84	3.32	*Ocotea* (Lauraceae)	26	1.76	*Lacistema* (Lacistemataceae)	2
14	*Swartzia* (Fabaceae)	0.266	1.79	2.17	*Qualea* (Vochysiaceae)	24	1.62	*Licania* (Chrysobalanaceae)	2
15	*Ilex* (Aquifoliaceae)	0.214	1.44	3.25	*Dendropanax* (Araliaceae)	21	1.42	*Protium* (Burseraceae)	2
16	*Casearia* (Salicaceae)	0.212	1.43	1.15	*Lacistema* (Lacistemataceae)	19	1.29	*Randia* (Rubiaceae)	2
17	*Myracrodruon* (Anacardiaceae)	0.174	1.17	0.68	*Tapirira* (Anacardiaceae)	19	1.29	*Sclerolobium* (Fabaceae)	2
18	*Terminalia* (Combretaceae)	0.168	1.13	0.41	Not identified	18	1.22	*Siparuna* (Siparunaceae)	2
19	*Astronium* (Anacardiaceae)	0.156	1.05	1.08	*Casearia* (Salicaceae)	17	1.15	*Tabebuia* (Bignoniaceae)	2
20	*Qualea* (Vochysiaceae)	0.143	0.96	1.62	*Eugenia* (Myrtaceae)	17	1.15	*Tapirira* (Anacardiaceae)	2
21	*Tapirira* (Anacardiaceae)	0.124	0.84	1.29	*Astronium* (Anacardiaceae)	16	1.08	*Vitex* (Lamiaceae)	2
22	*Virola* (Myristicaceae)	0.118	0.80	2.44	*Piptadenia* (Fabaceae)	16	1.08	*Acrocomia* (Arecaceae)	1
23	*Andira* (Fabaceae)	0.117	0.78	0.81	*Lithraea* (Anacardiaceae)	15	1.01	*Alchornea* (Euphorbiaceae)	1
24	*Acrocomia* (Arecaceae)	0.093	0.63	0.14	*Andira* (Fabaceae)	12	0.81	*Alibertia* (Rubiaceae)	1
25	*Licania* (Chrysobalanaceae)	0.089	0.60	0.68	*Alibertia* (Rubiaceae)	11	0.74	*Andira* (Fabaceae)	1
26	Not identified	0.087	0.59	1.22	*Licania* (Chrysobalanaceae)	10	0.68	*Apuleia* (Fabaceae)	1
27	*Peltophorum* (Fabaceae)	0.087	0.58	0.20	*Myracrodruon* (Anacardiaceae)	10	0.68	*Callisthene* (Vochysiaceae)	1
28	*Schefflera* (Araliaceae)	0.082	0.56	0.41	*Aspidosperma* (Apocynaceae)	6	0.41	*Casearia* (Salicaceae)	1
29	*Vernonia* (Asteraceae)	0.082	0.55	0.41	*Cedrela* (Meliaceae)	6	0.41	*Cedrela* (Meliaceae)	1
30	*Picramnia* (Picramniaceae)	0.082	0.55	0.14	*Dialium* (Fabaceae)	6	0.41	*Copaifera* (Fabaceae)	1
31	*Vitex* (Lamiaceae)	0.079	0.53	0.20	*Piper* (Piperaceae)	6	0.41	*Cupania* (Sapindaceae)	1
32	*Pera* (Euphorbiaceae)	0.075	0.50	0.27	*Schefflera* (Araliaceae)	6	0.41	*Dalbergia* (Fabaceae)	1
33	*Tabebuia* (Bignoniaceae)	0.070	0.47	0.41	*Tabebuia* (Bignoniaceae)	6	0.41	*Dialium* (Fabaceae)	1
34	*Sclerolobium* (Fabaceae)	0.069	0.46	0.07	*Terminalia* (Combretaceae)	6	0.41	*Diospyros* (Ebenaceae)	1
35	*Dialium* (Fabaceae)	0.062	0.42	0.41	*Vernonia* (Asteraceae)	6	0.41	*Guarea* (Meliaceae)	1
36	*Dendropanax* (Araliaceae)	0.061	0.41	1.42	*Cupania* (Sapindaceae)	5	0.34	*Guatteria* (Annonaceae)	1
37	*Eugenia* (Myrtaceae)	0.053	0.35	1.15	*Erythroxylum* (Erythroxylaceae)	5	0.34	*Guazuma* (Malvaceae)	1
38	*Rollinia* (Annonaceae)	0.043	0.29	0.14	*Guazuma* (Malvaceae)	5	0.34	*Guettarda* (Rubiaceae)	1
39	*Guazuma* (Malvaceae)	0.041	0.27	0.34	*Protium* (Burseraceae)	5	0.34	*Heteropterys* (Malpighiaceae)	1
40	*Pterogyne* (Fabaceae)	0.040	0.27	0.07	*Guatteria* (Annonaceae)	4	0.27	*Ixora* (Rubiaceae)	1
41	*Cupania* (Sapindaceae)	0.036	0.24	0.34	*Pera* (Euphorbiaceae)	4	0.27	*Lithraea* (Anacardiaceae)	1
42	*Aspidosperma* (Apocynaceae)	0.036	0.24	0.41	*Psidium* (Myrtaceae)	4	0.27	*Luehea* (Malvaceae)	1
43	*Luehea* (Malvaceae)	0.026	0.18	0.14	*Cassia* (Fabaceae)	3	0.20	*Marlieria* (Myrtaceae)	1
44	*Alibertia* (Rubiaceae)	0.026	0.18	0.74	*Marlieria* (Myrtaceae)	3	0.20	*Myracrodruon* (Anacardiaceae)	1
45	*Lacistema* (Lacistemataceae)	0.024	0.16	1.29	*Peltophorum* (Fabaceae)	3	0.20	*Nectandra* (Lauraceae)	1
46	*Cassia* (Fabaceae)	0.023	0.16	0.20	*Rudgea* (Rubiaceae)	3	0.20	Not identified	1
47	*Randia* (Rubiaceae)	0.022	0.15	0.07	*Vitex* (Lamiaceae)	3	0.20	*Ocotea* (Lauraceae)	1
48	*Guatteria* (Annonaceae)	0.022	0.15	0.27	*Acrocomia* (Arecaceae)	2	0.14	*Ouratea* (Ochnaceae)	1
49	*Senna* (Fabaceae)	0.021	0.14	0.14	*Heteropterys* (Malpighiaceae)	2	0.14	*Peltophorum* (Fabaceae)	1
50	*Protium* (Burseraceae)	0.020	0.13	0.34	*Luehea* (Malvaceae)	2	0.14	*Pera* (Euphorbiaceae)	1
51	*Plathymenia* (Fabaceae)	0.018	0.12	0.07	*Picramnia* (Picramniaceae)	2	0.14	*Picramnia* (Picramniaceae)	1
52	*Psidium* (Myrtaceae)	0.018	0.12	0.27	*Rollinia* (Annonaceae)	2	0.14	*Piper* (Piperaceae)	1
53	*Erythroxylum* (Erythroxylaceae)	0.013	0.09	0.34	*Senna* (Fabaceae)	2	0.14	*Piptadenia* (Fabaceae)	1
54	*Marlieria* (Myrtaceae)	0.012	0.08	0.20	*Trichilia* (Meliaceae)	2	0.14	*Plathymenia* (Fabaceae)	1
55	*Diospyros* (Ebenaceae)	0.009	0.06	0.07	*Alchornea* (Euphorbiaceae)	1	0.07	*Psidium* (Myrtaceae)	1
56	*Piper* (Piperaceae)	0.008	0.05	0.41	*Apuleia* (Fabaceae)	1	0.07	*Qualea* (Vochysiaceae)	1
57	*Guarea* (Meliaceae)	0.007	0.05	0.07	*Diospyros* (Ebenaceae)	1	0.07	*Rollinia* (Annonaceae)	1
58	*Ouratea* (Ochnaceae)	0.007	0.05	0.07	*Guarea* (Meliaceae)	1	0.07	*Rudgea* (Rubiaceae)	1
59	*Trichilia* (Meliaceae)	0.006	0.04	0.14	*Guettarda* (Rubiaceae)	1	0.07	*Schefflera* (Araliaceae)	1
60	*Heteropterys* (Malpighiaceae)	0.006	0.04	0.14	*Ixora* (Rubiaceae)	1	0.07	*Senna* (Fabaceae)	1
61	*Ixora* (Rubiaceae)	0.004	0.03	0.07	*Ouratea* (Ochnaceae)	1	0.07	*Swartzia* (Fabaceae)	1
62	*Apuleia* (Fabaceae)	0.004	0.02	0.07	*Plathymenia* (Fabaceae)	1	0.07	*Terminalia* (Combretaceae)	1
63	*Rudgea* (Rubiaceae)	0.003	0.02	0.20	*Pterogyne* (Fabaceae)	1	0.07	*Trichilia* (Meliaceae)	1
64	*Guettarda* (Rubiaceae)	0.001	0.01	0.07	*Randia* (Rubiaceae)	1	0.07	*Vernonia* (Asteraceae)	1
65	*Alchornea* (Euphorbiaceae)	0.001	0.01	0.07	*Sclerolobium* (Fabaceae)	1	0.07	*Virola* (Myristicaceae)	1

**Table 6.** Corrego Fundo Gallery Forest ranking by genus according to basal area (BA, including all multiple stems for each individual), number of individuals (N) and number of species (S), data from the third census.

**Rank**	**Genus**	**BA**	%**BA**	%**N**	**Genus**	**N**	%**N**	**Genus**	**S**
1	*Callisthene* (Vochysiaceae)	3.78	33.30	18.93	*Callisthene* (Vochysiaceae)	240	18.93	*Myrcia* (Myrtaceae)	5
2	*Copaifera* (Fabaceae)	1.35	11.94	3.86	*Siparuna* (Siparunaceae)	132	10.41	Not identified	5
3	*Terminalia* (Combretaceae)	0.77	6.82	2.68	*Myrcia* (Myrtaceae)	104	8.20	*Aspidosperma* (Apocynaceae)	4
4	*Tapirira* (Anacardiaceae)	0.59	5.18	4.10	*Licania* (Chrysobalanaceae)	74	5.84	*Eugenia* (Myrtaceae)	3
5	*Myrcia* (Myrtaceae)	0.54	4.78	8.20	*Tapirira* (Anacardiaceae)	52	4.10	*Machaerium* (Fabaceae)	3
6	*Pera* (Euphorbiaceae)	0.47	4.12	2.60	*Alibertia* (Rubiaceae)	50	3.94	*Casearia* (Salicaceae)	2
7	*Protium* (Burseraceae)	0.32	2.85	3.08	*Copaifera* (Fabaceae)	49	3.86	*Dalbergia* (Fabaceae)	2
8	*Siparuna* (Siparunaceae)	0.31	2.72	10.41	*Lacistema* (Lacistemataceae)	49	3.86	*Ixora* (Rubiaceae)	2
9	*Aspidosperma* (Apocynaceae)	0.27	2.39	2.05	*Protium* (Burseraceae)	39	3.08	*Licania* (Chrysobalanaceae)	2
10	*Dilodendron* (Sapindaceae)	0.27	2.36	2.37	*Terminalia* (Combretaceae)	34	2.68	*Matayba* (Sapindaceae)	2
11	*Xylopia* (Annonaceae)	0.26	2.29	1.81	*Pera* (Euphorbiaceae)	33	2.60	*Swartzia* (Fabaceae)	2
12	*Pseudobombax* (Malvaceae)	0.25	2.21	0.16	*Dilodendron* (Sapindaceae)	30	2.37	*Tabebuia* (Bignoniaceae)	2
13	*Licania* (Chrysobalanaceae)	0.24	2.11	5.84	*Eugenia* (Myrtaceae)	30	2.37	*Tapirira* (Anacardiaceae)	2
14	*Alibertia* (Rubiaceae)	0.17	1.48	3.94	*Virola* (Myristicaceae)	30	2.37	*Alibertia* (Rubiaceae)	1
15	*Virola* (Myristicaceae)	0.13	1.18	2.37	*Aspidosperma* (Apocynaceae)	26	2.05	*Andira* (Fabaceae)	1
16	*Astronium* (Anacardiaceae)	0.13	1.13	0.47	*Diospyros* (Ebenaceae)	25	1.97	*Apeiba* (Malvaceae)	1
17	*Diospyros* (Ebenaceae)	0.12	1.02	1.97	*Xylopia* (Annonaceae)	23	1.81	*Ardisia* (Primulaceae)	1
18	*Eugenia* (Myrtaceae)	0.11	0.93	2.37	*Tabebuia* (Bignoniaceae)	17	1.34	*Astronium* (Anacardiaceae)	1
19	*Tabebuia* (Bignoniaceae)	0.10	0.92	1.34	*Cupania* (Sapindaceae)	15	1.18	*Aureliana* (Solanaceae)	1
20	*Guazuma* (Malvaceae)	0.09	0.80	0.87	*Ixora* (Rubiaceae)	15	1.18	*Bowdichia* (Fabaceae)	1
21	*Cupania* (Sapindaceae)	0.07	0.63	1.18	*Swartzia* (Fabaceae)	15	1.18	*Byrsonima* (Malpighiaceae)	1
22	*Lacistema* (Lacistemataceae)	0.07	0.61	3.86	*Dendropanax* (Araliaceae)	11	0.87	*Callisthene* (Vochysiaceae)	1
23	*Apeiba* (Malvaceae)	0.07	0.59	0.24	*Guazuma* (Malvaceae)	11	0.87	*Calophyllum* (Calophyllaceae)	1
24	*Ixora* (Rubiaceae)	0.07	0.58	1.18	*Hirtella* (Chrysobalanaceae)	10	0.79	*Calyptranthes* (Myrtaceae)	1
25	Not identified	0.05	0.45	0.63	*Micropholis* (Sapotaceae)	10	0.79	*Campomanesia* (Myrtaceae)	1
26	*Andira* (Fabaceae)	0.05	0.45	0.63	*Andira* (Fabaceae)	8	0.63	*Cecropia* (Urticaceae)	1
27	*Vitex* (Lamiaceae)	0.05	0.43	0.63	*Ilex* (Aquifoliaceae)	8	0.63	*Copaifera* (Fabaceae)	1
28	*Campomanesia* (Myrtaceae)	0.04	0.39	0.24	*Schefflera* (Araliaceae)	8	0.63	*Cupania* (Sapindaceae)	1
29	*Dendropanax* (Araliaceae)	0.04	0.39	0.87	*Vitex* (Lamiaceae)	8	0.63	*Dendropanax* (Araliaceae)	1
30	*Micropholis* (Sapotaceae)	0.04	0.39	0.79	Not identified	8	0.63	*Dilodendron* (Sapindaceae)	1
31	*Schefflera* (Araliaceae)	0.04	0.38	0.63	*Casearia* (Salicaceae)	7	0.55	*Diospyros* (Ebenaceae)	1
32	*Lithraea* (Anacardiaceae)	0.04	0.36	0.39	*Machaerium* (Fabaceae)	7	0.55	*Duguetia* (Annonaceae)	1
33	*Eriotheca* (Malvaceae)	0.04	0.35	0.39	*Ouratea* (Ochnaceae)	7	0.55	*Endlicheria* (Lauraceae)	1
34	*Bowdichia* (Fabaceae)	0.04	0.35	0.24	*Astronium* (Anacardiaceae)	6	0.47	*Eriotheca* (Malvaceae)	1
35	*Machaerium* (Fabaceae)	0.03	0.30	0.55	*Eriotheca* (Malvaceae)	5	0.39	*Erythroxylum* (Erythroxylaceae)	1
36	*Hirtella* (Chrysobalanaceae)	0.03	0.27	0.79	*Lithraea* (Anacardiaceae)	5	0.39	*Guapira* (Nyctaginaceae)	1
37	*Swartzia* (Fabaceae)	0.03	0.26	1.18	*Aureliana* (Solanaceae)	4	0.32	*Guazuma* (Malvaceae)	1
38	*Casearia* (Salicaceae)	0.03	0.25	0.55	*Calyptranthes* (Myrtaceae)	4	0.32	*Guettarda* (Rubiaceae)	1
39	*Aureliana* (Solanaceae)	0.03	0.24	0.32	*Guapira* (Nyctaginaceae)	4	0.32	*Hirtella* (Chrysobalanaceae)	1
40	*Ouratea* (Ochnaceae)	0.02	0.22	0.55	*Matayba* (Sapindaceae)	4	0.32	*Ilex* (Aquifoliaceae)	1
41	*Luehea* (Malvaceae)	0.02	0.19	0.24	*Rapanea* (Primulaceae)	4	0.32	*Lacistema* (Lacistemataceae)	1
42	*Ilex* (Aquifoliaceae)	0.01	0.13	0.63	*Salacia* (Celastraceae)	4	0.32	*Lithraea* (Anacardiaceae)	1
43	*Matayba* (Sapindaceae)	0.01	0.12	0.32	*Apeiba* (Malvaceae)	3	0.24	*Luehea* (Malvaceae)	1
44	*Sterculia* (Malvaceae)	0.01	0.12	0.08	*Bowdichia* (Fabaceae)	3	0.24	*Micropholis* (Sapotaceae)	1
45	*Platypodium* (Fabaceae)	0.01	0.11	0.24	*Campomanesia* (Myrtaceae)	3	0.24	*Ocotea* (Lauraceae)	1
46	*Ocotea* (Lauraceae)	0.01	0.10	0.24	*Luehea* (Malvaceae)	3	0.24	*Ouratea* (Ochnaceae)	1
47	*Guapira* (Nyctaginaceae)	0.01	0.10	0.32	*Ocotea* (Lauraceae)	3	0.24	*Pera* (Euphorbiaceae)	1
48	*Rapanea* (Primulaceae)	0.01	0.09	0.32	*Platypodium* (Fabaceae)	3	0.24	*Piptadenia* (Fabaceae)	1
49	*Cecropia* (Urticaceae)	0.01	0.09	0.08	*Ardisia* (Primulaceae)	2	0.16	*Platypodium* (Fabaceae)	1
50	*Salacia* (Celastraceae)	0.01	0.08	0.32	*Byrsonima* (Malpighiaceae)	2	0.16	*Pouteria* (Sapotaceae)	1
51	*Dalbergia* (Fabaceae)	0.01	0.08	0.16	*Calophyllum* (Calophyllaceae)	2	0.16	*Protium* (Burseraceae)	1
52	*Calyptranthes* (Myrtaceae)	0.01	0.06	0.32	*Dalbergia* (Fabaceae)	2	0.16	*Pseudobombax* (Malvaceae)	1
53	*Byrsonima* (Malpighiaceae)	0.00	0.04	0.16	*Endlicheria* (Lauraceae)	2	0.16	*Rapanea* (Primulaceae)	1
54	*Pouteria* (Sapotaceae)	0.00	0.04	0.16	*Guettarda* (Rubiaceae)	2	0.16	*Rudgea* (Rubiaceae)	1
55	*Erythroxylum* (Erythroxylaceae)	0.00	0.03	0.08	*Pouteria* (Sapotaceae)	2	0.16	*Salacia* (Celastraceae)	1
56	*Duguetia* (Annonaceae)	0.00	0.03	0.08	*Pseudobombax* (Malvaceae)	2	0.16	*Schefflera* (Araliaceae)	1
57	*Guettarda* (Rubiaceae)	0.00	0.03	0.16	*Cecropia* (Urticaceae)	1	0.08	*Simarouba* (Simaroubaceae)	1
58	*Calophyllum* (Calophyllaceae)	0.00	0.02	0.16	*Duguetia* (Annonaceae)	1	0.08	*Siparuna* (Siparunaceae)	1
59	*Trichilia* (Meliaceae)	0.00	0.02	0.08	*Erythroxylum* (Erythroxylaceae)	1	0.08	*Sterculia* (Malvaceae)	1
60	*Endlicheria* (Lauraceae)	0.00	0.02	0.16	*Piptadenia* (Fabaceae)	1	0.08	*Terminalia* (Combretaceae)	1
61	*Rudgea* (Rubiaceae)	0.00	0.02	0.08	*Rudgea* (Rubiaceae)	1	0.08	*Trichilia* (Meliaceae)	1
62	*Ardisia* (Primulaceae)	0.00	0.02	0.16	*Simarouba* (Simaroubaceae)	1	0.08	*Virola* (Myristicaceae)	1
63	*Piptadenia* (Fabaceae)	0.00	0.01	0.08	*Sterculia* (Malvaceae)	1	0.08	*Vitex* (Lamiaceae)	1
64	*Xylosma* (Salicaceae)	0.00	0.01	0.08	*Trichilia* (Meliaceae)	1	0.08	*Xylopia* (Annonaceae)	1
65	*Simarouba* (Simaroubaceae)	0.00	0.01	0.08	*Xylosma* (Salicaceae)	1	0.08	*Xylosma* (Salicaceae)	1

**Table 7.** Corrego Fazendinha Gallery Forest ranking by species accoridng to basal area (BA) and number of individuals (N), data from the third census.

**Rank**	**Species**	**BA**	%**BA**	%**N**	**Species**	**N**	%**N**
1	*Callisthene major* Mart. (Vochysiaceae)	5.07	34.12	21.11	*Callisthene major* Mart. (Vochysiaceae)	312	21.11
2	*Copaifera langsdorffii* Desf. (Fabaceae)	0.95	6.43	2.98	*Siparuna guianensis* Aubl. (Siparunaceae)	274	18.54
3	*Piptadenia gonoacantha* (Mart.) J.F.Macbr. (Fabaceae)	0.69	4.62	1.08	*Campomanesia xanthocarpa* (Mart.) O.Berg (Myrtaceae)	94	6.36
4	*Siparuna guianensis* Aubl. (Siparunaceae)	0.65	4.40	18.54	*Myrcia tomentosa* (Aubl.) DC. (Myrtaceae)	61	4.13
5	*Myrcia tomentosa* (Aubl.) DC. (Myrtaceae)	0.60	4.05	4.13	*Dalbergia brasiliensis* Vogel (Fabaceae)	49	3.32
6	*Ocotea corymbosa* (Meisn.) Mez (Lauraceae)	0.52	3.49	1.76	*Nectandra oppositifolia* Nees & Mart. (Lauraceae)	45	3.04
7	*Cedrela fissilis* Vell. (Meliaceae)	0.45	3.03	0.41	*Copaifera langsdorffii* Desf. (Fabaceae)	44	2.98
8	*Campomanesia xanthocarpa* (Mart.) O.Berg (Myrtaceae)	0.43	2.88	6.36	*Ilex cerasifolia* Reissek (Aquifoliaceae)	41	2.77
9	*Nectandra oppositifolia* Nees & Mart. (Lauraceae)	0.39	2.64	3.04	*Myrcia splendens* (Sw.) DC. (Myrtaceae)	38	2.57
10	*Myrcia splendens* (Sw.) DC. (Myrtaceae)	0.37	2.46	2.57	*Virola sebifera* Aubl. (Myristicaceae)	36	2.44
11	*Lithraea molleoides* (Vell.) Engl. (Anacardiaceae)	0.33	2.25	1.01	*Swartzia* sp (Fabaceae)	31	2.10
12	*Machaerium villosum* Vogel (Fabaceae)	0.31	2.06	1.35	*Ocotea corymbosa* (Meisn.) Mez (Lauraceae)	26	1.76
13	*Dalbergia brasiliensis* Vogel (Fabaceae)	0.27	1.84	3.32	*Xylopia aromatica* (Lam.) Mart. (Annonaceae)	25	1.69
14	*Swartzia* sp (Fabaceae)	0.26	1.78	2.10	*Machaerium villosum* Vogel (Fabaceae)	20	1.35
15	*Xylopia aromatica* (Lam.) Mart. (Annonaceae)	0.24	1.63	1.69	*Dendropanax cuneatus* (DC.) Decne. & Planch. (Araliaceae)	20	1.35
16	*Casearia sylvestris* Sw. (Salicaceae)	0.21	1.43	1.15	*Tapirira guianensis* Aubl. (Anacardiaceae)	18	1.22
17	*Ilex cerasifolia* Reissek (Aquifoliaceae)	0.19	1.31	2.77	*Casearia sylvestris* Sw. (Salicaceae)	17	1.15
18	*Myracrodruon urundeuva* Allemão (Anacardiaceae)	0.17	1.17	0.68	*Piptadenia gonoacantha* (Mart.)J.F.Macbr. (Fabaceae)	16	1.08
19	*Tapirira guianensis* Aubl. (Anacardiaceae)	0.12	0.80	1.22	*Lacistema hasslerianum* Chodat (Lacistemataceae)	16	1.08
20	*Virola sebifera* Aubl. (Myristicaceae)	0.12	0.80	2.44	*Lithraea molleoides* (Vell.) Engl. (Anacardiaceae)	15	1.01
21	*Andira fraxinifolia* Benth. (Fabaceae)	0.12	0.78	0.81	*Astronium fraxinifolium* Schott (Anacardiaceae)	15	1.01
22	*Xylopia sericea* A.St.-Hil. (Annonaceae)	0.11	0.77	0.68	*Qualea grandiflora* Mart. (Vochysiaceae)	14	0.95
23	*Astronium fraxinifolium* Schott (Anacardiaceae)	0.10	0.65	1.01	Myrtaceae sp (Myrtaceae)	13	0.88
24	*Acrocomia aculeata* (Jacq.) Lodd. ex Mart. (Arecaceae)	0.09	0.63	0.14	*Eugenia florida* DC. (Myrtaceae)	12	0.81
25	*Machaerium isadelphum* (E.Mey.)Standl. (Fabaceae)	0.09	0.63	0.27	*Andira fraxinifolia* Benth. (Fabaceae)	12	0.81
26	*Peltophorum dubium* (Spreng.) Taub. (Fabaceae)	0.09	0.58	0.20	*Alibertia edulis* (Rich.) A.Rich. ex DC. (Rubiaceae)	11	0.74
27	*Terminalia argentea* Mart. (Combretaceae)	0.09	0.57	0.07	*Xylopia sericea* A.St.-Hil. (Annonaceae)	10	0.68
28	*Schefflera morototoni* (Aubl.) Maguire, Steyerm. & Frodin (Araliaceae)	0.08	0.56	0.41	*Myracrodruon urundeuva* Allemão (Anacardiaceae)	10	0.68
29	*Terminalia glabrescens* Mart. (Combretaceae)	0.08	0.55	0.34	*Ilex* sp (Aquifoliaceae)	7	0.47
30	*Vernonia* sp (Asteraceae)	0.08	0.55	0.41	*Vernonia* sp (Asteraceae)	6	0.41
31	*Picramnia parvifolia* Engl. (Picramniaceae)	0.08	0.55	0.14	*Schefflera morototoni* (Aubl.) Maguire, Steyerm. & Frodin (Araliaceae)	6	0.41
32	*Qualea grandiflora* Mart. (Vochysiaceae)	0.08	0.54	0.95	*Piper arboreum* Aubl. (Piperaceae)	6	0.41
33	*Vitex sellowiana* Cham. (Lamiaceae)	0.08	0.53	0.20	*Licania kunthiana* Hook.f. (Chrysobalanaceae)	6	0.41
34	*Pera glabrata* (Schott) Poepp. ex Baill. (Euphorbiaceae)	0.07	0.50	0.27	*Handroanthus ochraceus* (Cham.) Mattos (Bignoniaceae)	6	0.41
35	*Handroanthus ochraceus* (Cham.) Mattos (Bignoniaceae)	0.07	0.47	0.41	*Dialium* sp (Fabaceae)	6	0.41
36	*Sclerolobium paniculatum* Vogel (Fabaceae)	0.07	0.46	0.07	*Cedrela fissilis* Vell. (Meliaceae)	6	0.41
37	Myrtaceae sp (Myrtaceae)	0.07	0.45	0.88	*Terminalia glabrescens* Mart. (Combretaceae)	5	0.34
38	*Dialium* sp (Fabaceae)	0.06	0.42	0.41	*Qualea multiflora* Mart. (Vochysiaceae)	5	0.34
39	*Dendropanax cuneatus* (DC.) Decne. & Planch. (Araliaceae)	0.06	0.40	1.35	*Protium heptaphyllum* (Aubl.) Marchand (Burseraceae)	5	0.34
40	*Astronium* sp (Anacardiaceae)	0.06	0.40	0.07	*Guazuma ulmifolia* Lam. (Malvaceae)	5	0.34
41	*Licania kunthiana* Hook.f. (Chrysobalanaceae)	0.06	0.39	0.41	*Cupania vernalis* Cambess. (Sapindaceae)	5	0.34
42	*Eugenia florida* DC. (Myrtaceae)	0.04	0.29	0.81	*Qualea* sp (Vochysiaceae)	4	0.27
43	*Machaerium nyctitans* (Fabaceae)	0.04	0.28	0.14	*Pera glabrata* (Schott) Poepp. ex Baill. (Euphorbiaceae)	4	0.27
44	*Guazuma ulmifolia* Lam. (Malvaceae)	0.04	0.27	0.34	*Machaerium isadelphum* (E.Mey.)Standl. (Fabaceae)	4	0.27
45	*Pterogyne* sp (Fabaceae)	0.04	0.27	0.07	*Licania* sp (Chrysobalanaceae)	4	0.27
46	*Cupania vernalis* Cambess. (Sapindaceae)	0.04	0.24	0.34	*Guatteria sellowiana* Schltdl. (Annonaceae)	4	0.27
47	*Qualea* sp (Vochysiaceae)	0.03	0.22	0.27	*Erythroxylum pelleterianum* A.St.-Hil. (Erythroxylaceae)	4	0.27
48	*Licania* sp (Chrysobalanaceae)	0.03	0.21	0.27	*Vitex sellowiana* Cham. (Lamiaceae)	3	0.20
49	*Rollinia laurifolia* Schltdl. (Annonaceae)	0.03	0.21	0.07	*Rudgea viburnoides* (Cham.) Benth. (Rubiaceae)	3	0.20
50	*Luehea grandiflora* Mart. (Malvaceae)	0.03	0.18	0.14	*Psidium guajava* L. (Myrtaceae)	3	0.20
51	*Alibertia edulis* (Rich.) A.Rich. ex DC. (Rubiaceae)	0.03	0.18	0.74	*Peltophorum dubium* (Spreng.) Taub. (Fabaceae)	3	0.20
52	*Randia armata* (Sw.) DC. (Rubiaceae)	0.02	0.15	0.07	Not identified 1	3	0.20
53	*Guatteria sellowiana* Schltdl. (Annonaceae)	0.02	0.15	0.27	*Marlieria* sp (Myrtaceae)	3	0.20
54	*Machaerium opacum* Vogel (Fabaceae)	0.02	0.14	0.14	*Lacistema* sp (Lacistemataceae)	3	0.20
55	*Aspidosperma olivaceum* Müll. Arg. (Apocynaceae)	0.02	0.14	0.20	*Eugenia dysenterica* DC. (Myrtaceae)	3	0.20
56	*Ilex* sp (Aquifoliaceae)	0.02	0.13	0.47	*Aspidosperma olivaceum* Müll.Arg. (Apocynaceae)	3	0.20
57	*Protium heptaphyllum* (Aubl.) Marchand (Burseraceae)	0.02	0.13	0.34	*Trichilia pallida* Sw. (Meliaceae)	2	0.14
58	*Lacistema hasslerianum* Chodat (Lacistemataceae)	0.02	0.13	1.08	*Picramnia parvifolia* Engl. (Picramniaceae)	2	0.14
59	*Plathymenia reticulata* Benth. (Fabaceae)	0.02	0.12	0.07	*Myrcia* sp1 (Myrtaceae)	2	0.14
60	*Cassia ferruginea* (Schrad.)DC. (Fabaceae)	0.02	0.12	0.14	*Myrcia multiflora* (Lam.) DC. (Myrtaceae)	2	0.14
61	*Qualea multiflora* Mart. (Vochysiaceae)	0.02	0.12	0.34	*Myrcia guianensis* (Aubl.) DC. (Myrtaceae)	2	0.14
62	*Campomanesia velutina* (Cambess.) O.Berg (Myrtaceae)	0.02	0.11	0.14	*Machaerium opacum* Vogel (Fabaceae)	2	0.14
63	*Myrcia multiflora* (Lam.) DC. (Myrtaceae)	0.02	0.11	0.14	*Machaerium nyctitans* (Fabaceae)	2	0.14
64	*Psidium guajava* L. (Myrtaceae)	0.01	0.09	0.20	*Luehea grandiflora* Mart. (Malvaceae)	2	0.14
65	Qualea multiflora subsp. pubescens (Mart.) Stafleu (Vochysiaceae)	0.01	0.08	0.07	*Heteropterys byrsonimifolia* A.Juss. (Malpighiaceae)	2	0.14
66	*Rollinia* sp (Annonaceae)	0.01	0.08	0.07	*Eugenia* sp (Myrtaceae)	2	0.14
67	*Marlieria* sp (Myrtaceae)	0.01	0.08	0.20	*Cassia ferruginea* (Schrad.)DC. (Fabaceae)	2	0.14
68	Not identified 2	0.01	0.08	0.07	*Campomanesia velutina* (Cambess.) O.Berg (Myrtaceae)	2	0.14
69	*Senna* sp2 (Fabaceae)	0.01	0.07	0.07	*Acrocomia aculeata* (Jacq.) Lodd. ex Mart. (Arecaceae)	2	0.14
70	*Senna* sp1 (Fabaceae)	0.01	0.07	0.07	*Terminalia argentea* Mart. (Combretaceae)	1	0.07
71	*Aspidosperma* sp1 (Apocynaceae)	0.01	0.06	0.07	*Tapirira obtusa* (Benth.) J.D.Mitch. (Anacardiaceae)	1	0.07
72	*Diospyros* sp (Ebenaceae)	0.01	0.06	0.07	*Swartzia myrtifolia* Sm. (Fabaceae)	1	0.07
73	*Myrcia rufipes* DC. (Myrtaceae)	0.01	0.05	0.07	*Senna* sp2 (Fabaceae)	1	0.07
74	*Piper arboreum* Aubl. (Piperaceae)	0.01	0.05	0.41	*Senna* sp1 (Fabaceae)	1	0.07
75	*Erythroxylum pelleterianum* A.St.-Hil. (Erythroxylaceae)	0.01	0.05	0.27	*Sclerolobium paniculatum* Vogel (Fabaceae)	1	0.07
76	*Guarea* sp (Meliaceae)	0.01	0.05	0.07	Rubiaceae sp (Rubiaceae)	1	0.07
77	*Ouratea* sp (Ochnaceae)	0.01	0.05	0.07	*Rollinia* sp (Annonaceae)	1	0.07
78	*Trichilia pallida* Sw. (Meliaceae)	0.01	0.04	0.14	*Rollinia laurifolia* Schltdl. (Annonaceae)	1	0.07
79	*Eugenia dysenterica* DC. (Myrtaceae)	0.01	0.04	0.20	*Randia armata* (Sw.) DC. (Rubiaceae)	1	0.07
80	*Myrcia* sp1 (Myrtaceae)	0.01	0.04	0.14	Qualea multiflora subsp. pubescens (Mart.) Stafleu (Vochysiaceae)	1	0.07
81	*Heteropterys byrsonimifolia* A.Juss. (Malpighiaceae)	0.01	0.04	0.14	*Pterogyne* sp (Fabaceae)	1	0.07
82	*Erythroxylum citrifolium* A.St.-Hil. (Erythroxylaceae)	0.01	0.04	0.07	*Psidium rufum* Mart. ex DC. (Myrtaceae)	1	0.07
83	*Cassia* sp (Fabaceae)	0.00	0.03	0.07	*Plathymenia reticulata* Benth. (Fabaceae)	1	0.07
84	*Tapirira obtusa* (Benth.) J.D.Mitch. (Anacardiaceae)	0.00	0.03	0.07	*Ouratea* sp (Ochnaceae)	1	0.07
85	Not identified 1	0.00	0.03	0.20	Not identified 2	1	0.07
86	*Lacistema* sp (Lacistemataceae)	0.00	0.03	0.20	*Myrcia* sp2 (Myrtaceae)	1	0.07
87	*Myrcia guianensis* (Aubl.) DC. (Myrtaceae)	0.00	0.03	0.14	*Myrcia rufipes* DC. (Myrtaceae)	1	0.07
88	*Eugenia* sp (Myrtaceae)	0.00	0.03	0.14	*Machaerium* sp (Fabaceae)	1	0.07
89	*Psidium rufum* Mart. ex DC. (Myrtaceae)	0.00	0.03	0.07	*Ixora gardneriana* Benth. (Rubiaceae)	1	0.07
90	*Aspidosperma subincanum* Mart. ex A.DC. (Apocynaceae)	0.00	0.03	0.07	*Guettarda viburnoides* Cham. & Schltdl. (Rubiaceae)	1	0.07
91	*Ixora gardneriana* Benth. (Rubiaceae)	0.00	0.03	0.07	*Guarea* sp (Meliaceae)	1	0.07
92	*Rubiaceae* sp (Rubiaceae)	0.00	0.03	0.07	*Erythroxylum citrifolium* A.St.-Hil. (Erythroxylaceae)	1	0.07
93	*Apuleia leiocarpa* (Vogel) J.F.Macbr. (Fabaceae)	0.00	0.02	0.07	*Diospyros* sp (Ebenaceae)	1	0.07
94	*Rudgea viburnoides* (Cham.) Benth. (Rubiaceae)	0.00	0.02	0.20	*Dendropanax* sp (Araliaceae)	1	0.07
95	*Myrcia* sp2 (Myrtaceae)	0.00	0.02	0.07	*Cassia* sp (Fabaceae)	1	0.07
96	*Aspidosperma* sp2 (Apocynaceae)	0.00	0.02	0.07	*Astronium* sp (Anacardiaceae)	1	0.07
97	*Machaerium* sp (Fabaceae)	0.00	0.01	0.07	*Aspidosperma subincanum* Mart. ex A.DC. (Apocynaceae)	1	0.07
98	*Guettarda viburnoides* Cham. & Schltdl. (Rubiaceae)	0.00	0.01	0.07	*Aspidosperma* sp2 (Apocynaceae)	1	0.07
99	*Dendropanax* sp (Araliaceae)	0.00	0.01	0.07	*Aspidosperma* sp1 (Apocynaceae)	1	0.07
100	Alchornea glandulosa subsp. iricurana (Casar.) Secco (Euphorbiaceae)	0.00	0.01	0.07	*Apuleia leiocarpa* (Vogel) J.F.Macbr. (Fabaceae)	1	0.07
101	*Swartzia myrtifolia* Sm. (Fabaceae)	0.00	0.01	0.07	Alchornea glandulosa subsp. iricurana (Casar.) Secco (Euphorbiaceae)	1	0.07

**Table 8.** Corrego Fundo Gallery Forest ranking by species according to basal area (BA) and number of individuals (N), data from the third census.

**Rank**	**Species**	**BA**	%**BA**	%**N**	**Species**	**N**	%**N**
1	*Callisthene major* Mart. (Vochysiaceae)	3.78	33.30	18.93	*Callisthene major* Mart. (Vochysiaceae)	240	18.93
2	*Copaifera langsdorffii* Desf. (Fabaceae)	1.35	11.94	3.86	*Siparuna guianensis* Aubl. (Siparunaceae)	132	10.41
3	*Terminalia glabrescens* Mart. (Combretaceae)	0.77	6.82	2.68	*Licania kunthiana* Hook.f. (Chrysobalanaceae)	73	5.76
4	*Tapirira guianensis* Aubl. (Anacardiaceae)	0.57	5.04	3.86	*Myrcia rostrata* DC. (Myrtaceae)	51	4.02
5	*Pera glabrata* (Schott) Poepp. ex Baill. (Euphorbiaceae)	0.47	4.12	2.60	*Alibertia edulis* (Rich.) A.Rich. ex DC. (Rubiaceae)	50	3.94
6	*Protium heptaphyllum* (Aubl.) Marchand (Burseraceae)	0.32	2.85	3.08	*Copaifera langsdorffii* Desf. (Fabaceae)	49	3.86
7	*Siparuna guianensis* Aubl. (Siparunaceae)	0.31	2.72	10.41	*Lacistema hasslerianum* Chodat (Lacistemataceae)	49	3.86
8	*Myrcia rostrata* DC. (Myrtaceae)	0.28	2.50	4.02	*Tapirira guianensis* Aubl. (Anacardiaceae)	49	3.86
9	*Dilodendron bipinnatum* Radlk. (Sapindaceae)	0.27	2.36	2.37	*Protium heptaphyllum* (Aubl.) Marchand (Burseraceae)	39	3.08
10	*Xylopia aromatica* (Lam.) Mart. (Annonaceae)	0.26	2.29	1.81	*Terminalia glabrescens* Mart. (Combretaceae)	34	2.68
11	*Pseudobombax tomentosum* (Mart. & Zucc.) A.Robyns (Malvaceae)	0.25	2.21	0.16	*Pera glabrata* (Schott) Poepp. ex Baill. (Euphorbiaceae)	33	2.60
12	*Licania kunthiana* Hook.f. (Chrysobalanaceae)	0.24	2.10	5.76	*Dilodendron bipinnatum* Radlk. (Sapindaceae)	30	2.37
13	*Alibertia edulis* (Rich.) A.Rich. ex DC. (Rubiaceae)	0.17	1.48	3.94	*Virola sebifera* Aubl. (Myristicaceae)	30	2.37
14	*Virola sebifera* Aubl. (Myristicaceae)	0.13	1.18	2.37	*Diospyros brasiliensis* Mart. ex Miq. (Ebenaceae)	25	1.97
15	*Astronium fraxinifolium* Schott (Anacardiaceae)	0.13	1.13	0.47	*Myrcia guianensis* (Aubl.) DC. (Myrtaceae)	24	1.89
16	*Diospyros brasiliensis* Mart. ex Miq. (Ebenaceae)	0.12	1.02	1.97	*Xylopia aromatica* (Lam.) Mart. (Annonaceae)	23	1.81
17	*Aspidosperma darienense* Woodson ex Dwyer (Apocynaceae)	0.10	0.90	1.10	*Myrcia* sp. (Myrtaceae)	17	1.34
18	*Aspidosperma subincanum* Mart. ex A.DC. (Apocynaceae)	0.10	0.86	0.24	*Eugenia* sp. (Myrtaceae)	16	1.26
19	*Tabebuia serratifolia* (Vahl) G. Nicholson (Bignoniaceae)	0.10	0.84	1.26	*Tabebuia serratifolia* (Vahl) G. Nicholson (Bignoniaceae)	16	1.26
20	*Guazuma ulmifolia* Lam. (Malvaceae)	0.09	0.80	0.87	*Cupania vernalis* Cambess. (Sapindaceae)	15	1.18
21	*Myrcia* sp. (Myrtaceae)	0.09	0.78	1.34	*Aspidosperma darienense* Woodson ex Dwyer (Apocynaceae)	14	1.10
22	*Myrcia tomentosa* (Aubl.) DC. (Myrtaceae)	0.09	0.77	0.87	*Ixora gardneriana* Benth. (Rubiaceae)	14	1.10
23	*Myrcia guianensis* (Aubl.) DC. (Myrtaceae)	0.08	0.71	1.89	*Swartzia* sp. (Fabaceae)	14	1.10
24	*Cupania vernalis* Cambess. (Sapindaceae)	0.07	0.63	1.18	*Eugenia florida* DC. (Myrtaceae)	13	1.03
25	*Lacistema hasslerianum* Chodat (Lacistemataceae)	0.07	0.61	3.86	*Dendropanax cuneatus* (DC.) Decne. & Planch. (Araliaceae)	11	0.87
26	*Apeiba tibourbou* Aubl. (Malvaceae)	0.07	0.59	0.24	*Guazuma ulmifolia* Lam. (Malvaceae)	11	0.87
27	*Ixora gardneriana* Benth. (Rubiaceae)	0.06	0.56	1.10	*Myrcia tomentosa* (Aubl.) DC. (Myrtaceae)	11	0.87
28	*Eugenia florida* DC. (Myrtaceae)	0.06	0.51	1.03	*Hirtella hebeclada* Moric. ex DC. (Chrysobalanaceae)	10	0.79
29	*Andira fraxinifolia* Benth. (Fabaceae)	0.05	0.45	0.63	*Micropholis gardneriana* (A.DC.) Pierre (Sapotaceae)	10	0.79
30	*Vitex polygama* Cham. (Lamiaceae)	0.05	0.43	0.63	*Andira fraxinifolia* Benth. (Fabaceae)	8	0.63
31	*Eugenia* sp. (Myrtaceae)	0.05	0.41	1.26	*Ilex cerasifolia* Reissek (Aquifoliaceae)	8	0.63
32	*Campomanesia* sp. (Myrtaceae)	0.04	0.39	0.24	*Schefflera morototoni* (Aubl.) Maguire, Steyerm. & Frodin (Araliaceae)	8	0.63
33	*Aspidosperma cylindrocarpon* Müll.Arg. (Apocynaceae)	0.04	0.39	0.47	*Vitex polygama* Cham. (Lamiaceae)	8	0.63
34	*Dendropanax cuneatus* (DC.) Decne. & Planch. (Araliaceae)	0.04	0.39	0.87	*Ouratea castaneifolia* (DC.) Engl. (Ochnaceae)	7	0.55
35	*Micropholis gardneriana* (A.DC.) Pierre (Sapotaceae)	0.04	0.39	0.79	*Aspidosperma cylindrocarpon* Müll.Arg. (Apocynaceae)	6	0.47
36	*Schefflera morototoni* (Aubl.) Maguire, Steyerm. & Frodin (Araliaceae)	0.04	0.38	0.63	*Astronium fraxinifolium* Schott (Anacardiaceae)	6	0.47
37	*Lithraea molleoides* (Vell.) Engl. (Anacardiaceae)	0.04	0.36	0.39	*Casearia sylvestris* Sw. (Salicaceae)	5	0.39
38	*Eriotheca candolleana* (K.Schum.) A.Robyns (Malvaceae)	0.04	0.35	0.39	*Eriotheca candolleana* (K.Schum.) A.Robyns (Malvaceae)	5	0.39
39	*Banisteriopsis anisandra* (A.Juss.) B.Gates (Malpighiaceae)	0.04	0.35	0.24	*Lithraea molleoides* (Vell.) Engl. (Anacardiaceae)	5	0.39
40	*Hirtella hebeclada* Moric. ex DC. (Chrysobalanaceae)	0.03	0.27	0.79	*Aureliana velutina* Sendtn. (Solanaceae)	4	0.32
41	Myrtaceae 3	0.03	0.27	0.16	*Calyptranthes* sp. (Myrtaceae)	4	0.32
42	*Swartzia* sp. (Fabaceae)	0.03	0.25	1.10	*Guapira opposita* (Vell.) Reitz (Nyctaginaceae)	4	0.32
43	*Aureliana velutina* Sendtn. (Solanaceae)	0.03	0.24	0.32	*Machaerium villosum* Vogel (Fabaceae)	4	0.32
44	*Aspidosperma olivaceum* Müll.Arg. (Apocynaceae)	0.03	0.23	0.24	*Rapanea umbellata* (Mart.) Mez (Primulaceae)	4	0.32
45	*Ouratea castaneifolia* (DC.) Engl. (Ochnaceae)	0.02	0.22	0.55	*Salacia elliptica* (Mart.) G.Don (Celastraceae)	4	0.32
46	*Luehea grandiflora* Mart. (Malvaceae)	0.02	0.19	0.24	*Apeiba tibourbou* Aubl. (Malvaceae)	3	0.24
47	*Machaerium villosum* Vogel (Fabaceae)	0.02	0.17	0.32	*Aspidosperma olivaceum* Müll.Arg. (Apocynaceae)	3	0.24
48	*Casearia sylvestris* Sw. (Salicaceae)	0.02	0.16	0.39	*Aspidosperma subincanum* Mart. ex A.DC. (Apocynaceae)	3	0.24
49	*Tapirira obtusa* (Benth.) J.D.Mitch. (Anacardiaceae)	0.01	0.13	0.24	*Banisteriopsis anisandra* (A.Juss.) B.Gates (Malpighiaceae)	3	0.24
50	*Ilex cerasifolia* Reissek (Aquifoliaceae)	0.01	0.13	0.63	*Campomanesia* sp. (Myrtaceae)	3	0.24
51	*Sterculia striata* A. St.-Hil. & Naudin (Malvaceae)	0.01	0.12	0.08	*Luehea grandiflora* Mart. (Malvaceae)	3	0.24
52	*Machaerium nyctitans* (Vell.) Benth. (Fabaceae)	0.01	0.12	0.16	Myrtaceae 1	3	0.24
53	*Platypodium elegans* Vogel (Fabaceae)	0.01	0.11	0.24	*Ocotea corymbosa* (Meisn.) Mez (Lauraceae)	3	0.24
54	*Ocotea corymbosa* (Meisn.) Mez (Lauraceae)	0.01	0.10	0.24	*Platypodium elegans* Vogel (Fabaceae)	3	0.24
55	*Guapira opposita* (Vell.) Reitz (Nyctaginaceae)	0.01	0.10	0.32	*Tapirira obtusa* (Benth.) J.D.Mitch. (Anacardiaceae)	3	0.24
56	*Casearia gossypiosperma* Briq. (Salicaceae)	0.01	0.10	0.16	*Ardisia glauciflora* Urb. (Primulaceae)	2	0.16
57	*Rapanea umbellata* (Mart.) Mez (Primulaceae)	0.01	0.09	0.32	*Byrsonima sericea* DC. (Malpighiaceae)	2	0.16
58	Myrtaceae 1	0.01	0.09	0.24	*Calophyllum brasiliense* Cambess. (Calophyllaceae)	2	0.16
59	*Cecropia pachystachya* Trécul (Urticaceae)	0.01	0.09	0.08	*Casearia gossypiosperma* Briq. (Salicaceae)	2	0.16
60	*Salacia elliptica* (Mart.) G.Don (Celastraceae)	0.01	0.08	0.32	*Endlicheria paniculata* (Spreng.) J.F.Macbr. (Lauraceae)	2	0.16
61	*Tabebuia impetiginosa* (Mart. ex DC.) Standl. (Bignoniaceae)	0.01	0.08	0.08	*Guettarda viburnoides* Cham. & Schltdl. (Rubiaceae)	2	0.16
62	*Matayba floribunda* Radlk. (Sapindaceae)	0.01	0.07	0.16	*Machaerium nyctitans* (Vell.) Benth. (Fabaceae)	2	0.16
63	*Dalbergia brasiliensis* Vogel (Fabaceae)	0.01	0.07	0.08	*Matayba floribunda* Radlk. (Sapindaceae)	2	0.16
64	*Calyptranthes* sp. (Myrtaceae)	0.01	0.06	0.32	*Matayba guianensis* Aubl. (Sapindaceae)	2	0.16
65	*Matayba guianensis* Aubl. (Sapindaceae)	0.01	0.05	0.16	Myrtaceae 3	2	0.16
66	Rubiaceae 1	0.00	0.04	0.08	*Pouteria glomerata* (Miq.) Radlk. (Sapotaceae)	2	0.16
67	*Byrsonima sericea* DC. (Malpighiaceae)	0.00	0.04	0.16	*Pseudobombax tomentosum* (Mart. & Zucc.) A.Robyns (Malvaceae)	2	0.16
68	*Pouteria glomerata* (Miq.) Radlk. (Sapotaceae)	0.00	0.04	0.16	*Cecropia pachystachya* Trécul (Urticaceae)	1	0.08
69	*Erythroxylum daphnites* Mart. (Erythroxylaceae)	0.00	0.03	0.08	*Dalbergia brasiliensis* Vogel (Fabaceae)	1	0.08
70	Myrtaceae 4	0.00	0.03	0.08	*Dalbergia frutescens* (Vell.)Britton (Fabaceae)	1	0.08
71	*Duguetia lanceolata* A.St.-Hil. (Annonaceae)	0.00	0.03	0.08	*Duguetia lanceolata* A.St.-Hil. (Annonaceae)	1	0.08
72	*Guettarda viburnoides* Cham. & Schltdl. (Rubiaceae)	0.00	0.03	0.16	*Erythroxylum daphnites* Mart. (Erythroxylaceae)	1	0.08
73	*Calophyllum brasiliense* Cambess. (Calophyllaceae)	0.00	0.02	0.16	*Eugenia dodonaeifolia* Cambess. (Myrtaceae)	1	0.08
74	*Trichilia pallida* Sw. (Meliaceae)	0.00	0.02	0.08	Ixora cf. bahiensis (Rubiaceae)	1	0.08
75	*Endlicheria paniculata* (Spreng.) J.F.Macbr. (Lauraceae)	0.00	0.02	0.16	*Licania* sp. (Chrysobalanaceae)	1	0.08
76	Myrtaceae 2	0.00	0.02	0.08	*Machaerium brasiliense* Vogel (Fabaceae)	1	0.08
77	*Rudgea viburnoides* (Cham.) Benth. (Rubiaceae)	0.00	0.02	0.08	*Myrcia splendens* (Sw.) DC. (Myrtaceae)	1	0.08
78	*Ardisia glauciflora* Urb. (Primulaceae)	0.00	0.02	0.16	Myrtaceae 2	1	0.08
79	*Myrcia splendens* (Sw.) DC. (Myrtaceae)	0.00	0.02	0.08	Myrtaceae 4	1	0.08
80	Ixora cf. bahiensis (Rubiaceae)	0.00	0.01	0.08	*Piptadenia gonoacantha* (Mart.)J.F.Macbr. (Fabaceae)	1	0.08
81	*Licania* sp. (Chrysobalanaceae)	0.00	0.01	0.08	Rubiaceae 1	1	0.08
82	*Piptadenia gonoacantha* (Mart.)J.F.Macbr. (Fabaceae)	0.00	0.01	0.08	*Rudgea viburnoides* (Cham.) Benth. (Rubiaceae)	1	0.08
83	*Eugenia dodonaeifolia* Cambess. (Myrtaceae)	0.00	0.01	0.08	*Simarouba amara* Aubl. (Simaroubaceae)	1	0.08
84	*Xylosma prockia* (Turcz.) Turcz. (Salicaceae)	0.00	0.01	0.08	*Sterculia striata* A. St.-Hil. & Naudin (Malvaceae)	1	0.08
85	*Dalbergia frutescens* (Vell.)Britton (Fabaceae)	0.00	0.01	0.08	*Swartzia myrtifolia* Sm. (Fabaceae)	1	0.08
86	*Swartzia myrtifolia* Sm. (Fabaceae)	0.00	0.01	0.08	*Tabebuia impetiginosa* (Mart. ex DC.) Standl. (Bignoniaceae)	1	0.08
87	*Machaerium brasiliense* Vogel (Fabaceae)	0.00	0.01	0.08	*Trichilia pallida* Sw. (Meliaceae)	1	0.08
88	*Simarouba amara* Aubl. (Simaroubaceae)	0.00	0.01	0.08	*Xylosma prockia* (Turcz.) Turcz. (Salicaceae)	1	0.08

**Table 9.** Corrego Fazendinha and Corrego Fundo Gallery Forests tree Demographic Plot. BA is Basal Area.

**Period**	**Mortality Rate** [%/**yr**]	**Recruitment Rate [%/yr**]	**BA Losses** [**m^2^/ha/yr**]	**BA Gains** [**m^2^/ha/yr**]
Corrego Fazendinha				
2007 - 2011	0.93	2.62	0.51	1.96
2011 - 2015	5.19	1.66	1.6	2.39
Corrego Fundo				
2006 - 2010	2.43	1.5	2.56	1.52
2010 - 2014	2.82	0.85	2.28	1.08
